# Cysteamine Chemisorption at Mercury–Solution Interfaces in the Context of Redox and Microdissociation Equilibria

**DOI:** 10.1021/acs.langmuir.3c03744

**Published:** 2024-03-15

**Authors:** Vlastimil Dorčák, Ondřej Kroutil, Martin Kabeláč, Jiří Janata, Jan Vacek

**Affiliations:** † Department of Medical Chemistry and Biochemistry, Faculty of Medicine and Dentistry, Palacky University, Hnevotinska 3, Olomouc 775 15, Czech Republic; ‡ Central European Institute of Technology, Masaryk University, Kamenice 5, Brno 625 00, Czech Republic; § Department of Chemistry, Faculty of Science, University of South Bohemia, Branisovska 31, Ceske Budejovice 370 05, Czech Republic; ∥ School of Chemistry and Biochemistry, Georgia Institute of Technology, Atlanta, Georgia 30332-0400, United States

## Abstract

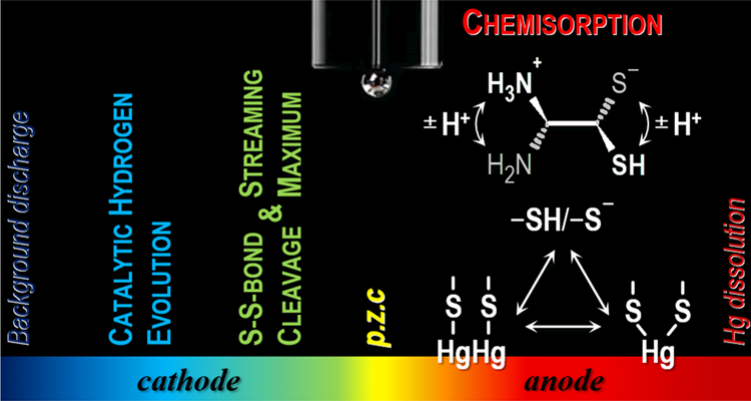

The redox behavior and chemisorption of cysteamine (CA) at a charged mercury surface are described, with an emphasis on its acid–base properties supported by molecular dynamics and quantum mechanical calculations. It was found that CA forms chemisorbed layers on the surface of the mercury electrode. The formation of Hg-CA complexes is connected to mercury disproportionation, as reflected in peaks SII and SI at potentials higher than the electrode potential of zero charge (p.z.c.). Both the process of chemisorption of CA and its consequent redox transformation are proton-dependent. Also, depending on the protonation of CA, the formation of typical populations of chemisorbed conformers can be observed. In addition, cystamine (CA disulfide dimer) can be reduced on the mercury surface. Between the potentials of this reduction and peak SI, the p.z.c. of the electrode used can be found. Furthermore, CA can serve as an LMW catalyst for hydrogen evolution. The mechanistic insights presented here can be used for follow-up research on CA chemisorption and targeted modification of other metallic surfaces.

## Introduction

Cysteamine (CA) is a relatively small molecule of biological and technological relevance, containing an amino group and a thiol group (H_2_N–CH_2_–CH_2_–SH). CA is produced endogenously^1^ and can act as a versatile therapeutic agent.^2^ Most of its biological effects can be explained by thiol/disulfide exchange reactions and scavenging electrophilic intermediates. However, CA can also form S–S bonds with cysteine (Cys) and its residues in proteins, thereby blocking/modifying their functionalities.

In technological applications, CA is chiefly utilized for the functionalization of various conductive surfaces, thus serving as a platform for the further attachment of biorecognition layers that enable the specific detection of desired analytes (reviewed in ref (3)). Other recent applications include, e.g., the covalent attachment of CA onto graphene oxide in the engineering of a selective and high-performance Hg(II) adsorbent,^4^ the controlled etching and tapering of Au nanorods by CA,^5^ the use of CA as a linker for the controlled self-assembly of Au nanorods,^6^ or the introduction of Cu-CA nanoparticles as a new type of radiosensitizer.^7^

CA and its disulfide group containing dimer, cystamine (CSS; H_2_N–CH_2_–CH_2_–S–S–CH_2_–CH_2_–NH_2_, see also Figure S1 in the Supporting Information), were previously determined polarographically at pH 7.4 using a CA anodic wave with a half-wave potential (*E*_1/2_) of −0.42 V (vs saturated calomel electrode) and a CSS cathodic wave with an *E*_1/2_ 0.2 V more negative.^8^ It was also shown that over a wide pH range, the anodic wave corresponds to the oxidation of the mercury electrode by CA thiol or thiolate group while the cathodic wave corresponds to the reduction of the CSS S–S bond.^9,10^ In other polarographic studies, CA and CSS were used to investigate systems containing cobalt(II) and Cys-like compounds in alkaline media.^11−13^

Recently, we utilized CA as a molecular probe to evaluate the reactivity of electrophilic compounds toward primary amine and thiol groups.^14^ Constant-current chronopotentiometric stripping (CPS) was used to measure CA responses at a hanging mercury drop electrode (HMDE). CA produced three well-developed reduction peaks. Analogous to Cys and/or Cys-containing peptides and proteins, the first two peaks were attributed to the two-step reduction of chemisorbed CA molecules on the electrode surface,^15,16^ whereas the third one was attributed to the involvement of CA in a catalytic hydrogen evolution reaction (CHER), reviewed in refs (17−19).

Herein, we investigated the electrochemical behavior of CA and CSS at an HMDE over its entire potential range in aqueous solutions using cyclic voltammetry and alternating current voltammetry (CV and ACV) and CPS. The obtained results are discussed in terms of CA acid–base properties^20^ and supported by previously parametrized molecular dynamics (MD) and quantum mechanics (QM) simulations at electrically charged mercury surfaces.^21^

## Experimental Section

### Reagents

Cysteamine hydrochloride, cystamine dihydrochloride, hexamine ruthenium(III) chloride, and chemicals and water (ACS reagents) for the preparation of buffer solutions were purchased from Sigma-Aldrich or Merck.

### Apparatus

Electrochemical measurements were performed using a μAutolab III analyzer (EcoChemie) connected to a VA-stand 663 (Metrohm) with the three-electrode setup, consisting of a hanging mercury drop electrode (HMDE) as the working electrode, a Ag|AgCl|3 M KCl electrode as the reference, and a glassy carbon rod as the auxiliary electrode. The pH measurements were done with an HI 2211 pH/ORP Meter (Hanna Instruments).

### Measurements

A cell thermostated at 25 °C was used for conventional *in situ* measurements. Argon was used to deaerate solutions before voltammetric measurements. Adsorptive transfer (AdT) *ex situ* experiments were conducted open to the air (unless stated otherwise) at a laboratory temperature. Aqueous solutions of 0.15 M Na-phosphate buffer (pH 6 and 7.7) and 0.15 M Britton–Robinson buffer (pH 2.1–11.8) served as supporting electrolytes. A constant ionic strength of the Britton–Robinson buffer was maintained with NaClO_4_.

### Procedures

Conventional measurements by CV, ACV, or CPS were performed after an accumulation time *t*_A_ at an accumulation potential *E*_A_ or at an open-circuit potential. Cyclic voltammograms were recorded at a scan rate ν of 0.5 V/s from an initial potential *E*_i_ to a vertex potential *E*_v_ (forward scan) and then back to *E*_i_ (backward scan). AC voltammograms were recorded in the cathodic direction at a 230 Hz frequency and a scan rate of 9 mV/s. Chronopotentiograms were recorded at a stripping current *I*_str_ intensity from *E*_i_ in the cathodic direction.

The AdT-CPS consisted of three steps: (i) CA was first accumulated for 60 s on the HMDE from a buffer solution at an open-circuit potential. (ii) Then, the CA-modified HMDE (CA-HMDE) was washed with an excess of a blank buffer solution. (iii) Finally, the washed CA-HMDE was immersed into the blank buffer solution to perform the CPS measurement. To avoid affecting the protonation equilibrium within the interface, the same composition of the buffer solution was used in all three steps (i, ii, and iii). With AdT-CV, in the last step (iii), the CA-HMDE was transferred into the buffer solution containing 5 mM [Ru(NH_3_)_6_]Cl_3_, and prior to the cyclic voltammetric measurement, the solution was deaerated with Ar.

#### Molecular Dynamics (MD) Simulations

##### Surface

As in our previous study,^21^ a rigid solid mercury surface was used systematically in all our simulations to simplify the simulation setup. It was shown by Bosio et al.^22^ and Böcker et al.^23^ that a liquid mercury surface can be replaced with a solid α-mercury lattice model. We have found (ref (21)) the Lennard-Jones parameters of Hg derived by Kuss et al.^24^ to be the ones that best reproduce the structure of water at a mercury|water interface, and we used them in this study as well.

##### Cysteamine

**Scheme 1:**
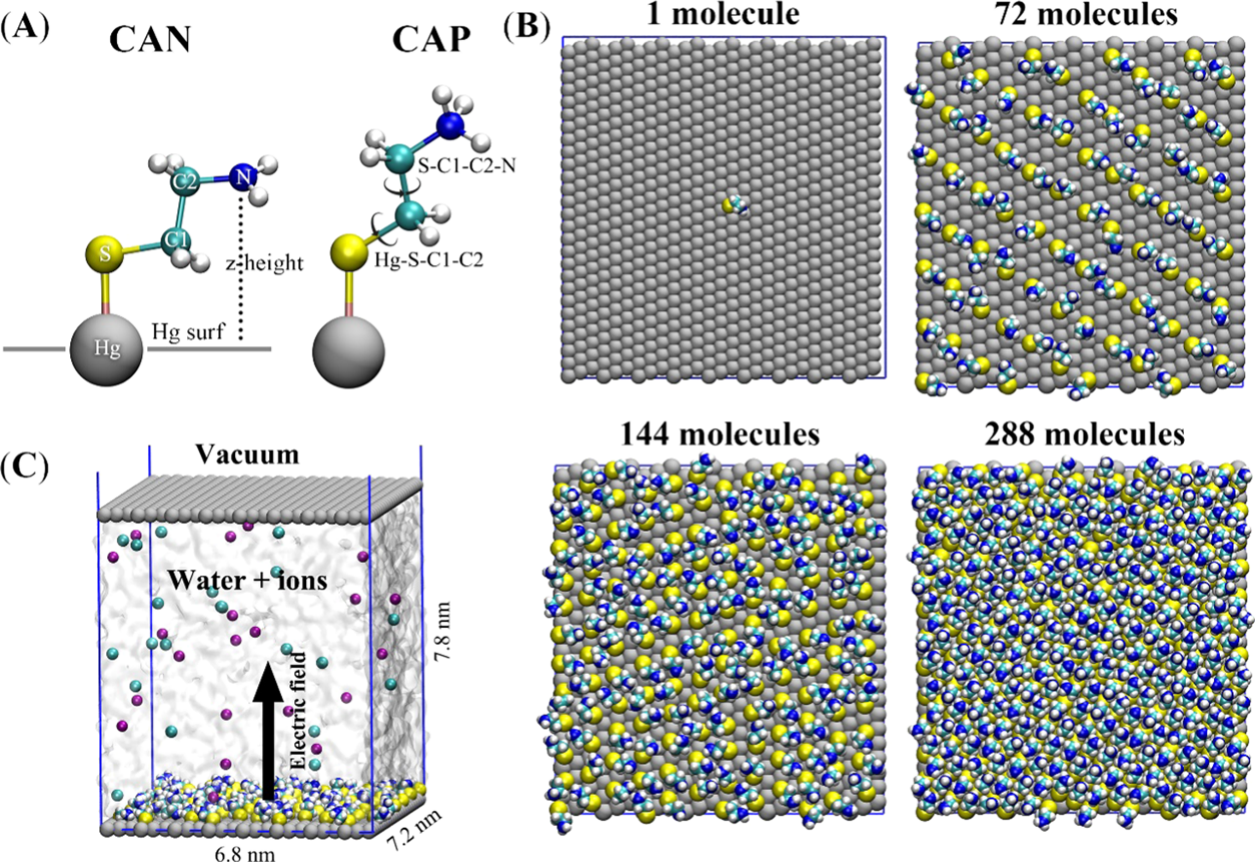
Molecular Dynamics (MD) Setup (A) Two forms of the simulated CA molecules and description of the variable used in Figures 3 and S4. (B) Top view of simulated systems with different surface coverage. (C) Side view of representative simulation box with highlighted dimensions of the system, its composition and application of the electric field.

A molecule(s) of CA was covalently anchored to the mercury surface *via* its thiol group (Scheme 1A). Since the mercury surface is liquid, and to further simplify our simulations, we only considered atop binding sites, while excluding other binding sites such as bridge, fcc, and hps found on solid metal surfaces.^25^ The bonding parameters between the thiol group of CA and the mercury surface were taken from the work of Hirano et al.,^26^ and partial charges were derived by a standard restrained electrostatic potential (RESP) routine.^27^ Two possible protonation states of CA were systematically employed (Scheme 1A): a neutral form with an NH_2_-capping group (designated as CAN in subsequent text and graphs) and a positively charged form with a protonated NH_3_^+^ group (named CAP in subsequent text and graphs). To evaluate the influence of CA surface density on its conformation, we prepared systems with 1, 72, 144, and 288 strands on a mercury substrate of the same size (Scheme 1B). These coverages correspond to surface access numbers of 3.39 × 10^–12^, 2.44 × 10^–10^, 4.88 × 10^–10^, and 9.75 × 10^–10^ mol/cm^2^ or to an available area of (*a*_P_) 49.04, 0.68, 0.34, and 0.17 nm^2^, respectively.

##### Solvent

Water was used as a solvent in all of the simulated systems. We used a simple point-charge (SPC/E)^28^ water model together with corresponding ion parameters by Joung and Cheatham^29^ in all simulations. We used 21 sodium ions and the same number of chloride ions in simulations with CAN molecules, representing a concentration of about 0.1 M. In the simulation with CAP molecules, we added the appropriate extra number of chloride ions to compensate for the positive charge on CAP molecules.

##### Simulation Cell

A scheme of simulated systems is depicted in Scheme 1C. All simulations were performed in a periodic box with dimensions of 6.8 × 7.2 × 100.0 nm for all of the systems. Water, ions, and CA molecules were confined between two solid monolayer mercury surfaces; one surface was positioned at the origin of the *z-*axis coordinate, and the second at a *z-*axis distance of 7.8 nm from the first mercury. The upper slab served to prevent the evaporation of water during the simulation into the vacuum part that filled the rest of the box in the larger *z*-axis dimension. The overall height of the simulation box was large enough to suppress the influence of the applied electric field on the periodic images of the box in the *z-*direction.

##### Application of Voltage

The mercury surface was influenced by applying a voltage between the two mercury surfaces of the periodic box (Scheme 1C). The sequence of voltages −25, 0, and +25 V was applied systematically in all of the simulations. The voltage was generated by setting up the appropriate electric field multiplied by the height of the gap between the two surfaces (7.5 nm). This corresponds to a common range of voltages used in theoretical studies of the effects of external electric fields on physisorption.^30,31^

##### MD Setup

The production runs in all 24 systems (corresponding to combinations of 2 protonation states of molecule, 4 surface densities, and 3 applied voltages) were 150 ns long. Three-dimensional particle mesh Ewald summation with correction for two-dimensional (2D) slabs^32^ was used to treat the long-range electrostatics. The v-rescale thermostat with a coupling time set to 1.0 ps maintained a temperature of 298 K throughout the whole production phase. Simulations were performed using the Gromacs 2021.4 software package.^33^ All analyses were performed with Gromacs tools and VMD software.^34^ For boxplot graphs, data from the final 10 ns were included in the analysis.

#### Quantum Mechanical Calculations

Two models were chosen to describe the conformational flexibility of CA on the mercury surface. The first consisted of CA bound to an isolated mercury atom, while in the second case, the system contained a cluster of nine atoms of a mercury slab in the same rigid geometry as used in the MD simulations. Similar to the MD computer experiments, two forms of CA were considered, with a protonated and a nonprotonated amino group.

CA potential energy surface for the first model was obtained as a two-dimensional dihedral profile of Hg–S–C1-C2 vs S–C1–C2-N torsion angles (Scheme 1), with the rest of the molecule relaxed. The values of both torsion angles were varied by 15° during the procedure.

For the second model, the calculations were restricted to combinations of key values of the above torsion angles, i.e., ± *gauche* and *trans* (i.e., 60, −60, and 180°).

The calculations were obtained at the density-functional theory (DFT) level using the B3LYP functional. The optimizations were performed with the def2-SVPP basis set, and the final energy was obtained using the same method as that with the def2-QZVPP basis set. The missing dispersion energy in the DFT theory was compensated for using Grimme’s empirical dispersion term GD3.^35^ The effect of hydration was included implicitly using the PCM model,^36^ and all DFT calculations were performed in the program Gaussian16.^37^

## Results and Discussion

**Figure 1:**
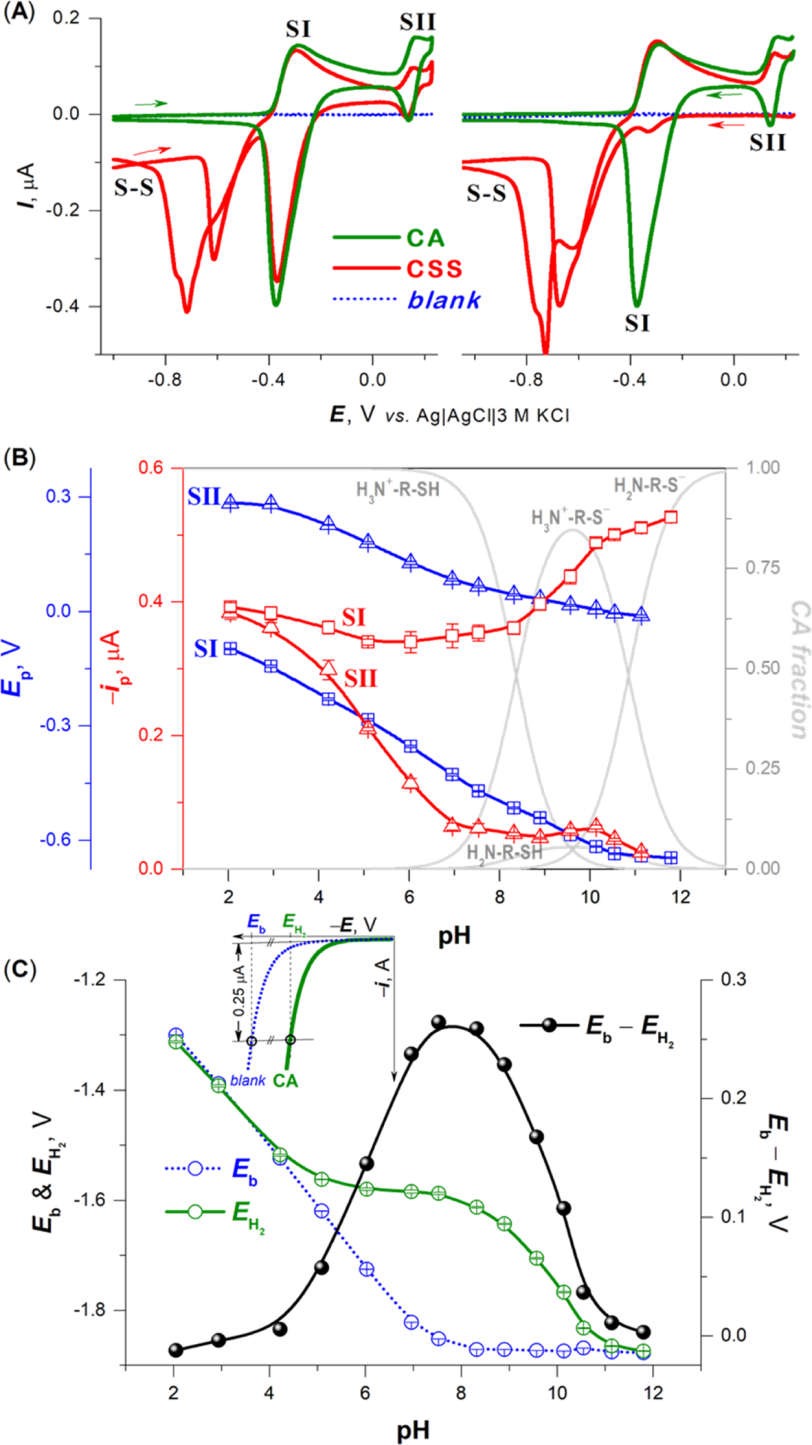
(A) Cyclic voltammograms of 100 μM CA and CSS at HMDE in 0.15 M Na-phosphate buffer, pH 6. Arrows indicate the direction of HMDE polarization in forward scans. Left: recorded from −1.0 to 0.23 V (forward anodic scan) and then back to −1.0 V (backward cathodic scan). Right: recorded from 0.23 to −1.8 V (forward cathodic scan) and then back to 0.23 V (backward anodic scan). (B) pH dependences of cathodic peak SI and SII potential (*E*_p_, blue) and current (*i*_p_, red) of 100 μM CA in 0.15 M Britton–Robinson buffer (with constant ionic strength) and pH distribution of the fractionally ionized forms of CA (gray) calculated from the micro-constants.^20^ Cyclic voltammograms were recorded from an initial potential *E*_i_ close to the potentials of the electrolytic dissolution of the electrode mercury to a vertex potential *E*_v_ at potentials of the supporting electrolyte discharge (forward cathodic scan) and then back to *E*_i_ (backward anodic scan). (C) pH dependences of *E*_b_ and *E*_H_2__values limiting the extent of HMDE polarization in the cathodic direction due to supporting electrolyte discharge and the CA involvement in CHER, respectively. The inset shows how the *E*_b_ and *E*_H_2__values were acquired from forward cathodic scans. Experimental points with error bars are averages with standard deviations of three measurements in the same solution.

Using CV with HMDE, we investigated the ability of CA and CSS to oxidize electrode mercury in an aqueous 0.15 M Na-phosphate buffer solution of pH 6. In accordance with the previous reports on Cys and cystine,^16,38^ two anodic (oxidation) peaks in the forward scan were observed, with the corresponding cathodic (reduction) counter peaks in the backward scan, indicating reversible redox processes (Figure 1A, left). The first anodic peak SI, at potentials close to the potential of zero charge (p.z.c.), is due to the oxidative formation of a CA compound with dimeric monovalent (electrode) mercury (Hg_2_^2+^), CA mercurous thiolate Hg_2_CA_2_:
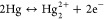
1
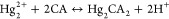
2

The second anodic peak SII, at potentials close to the electrolytic dissolution of the electrode mercury, is due to the oxidative formation of a CA compound with the bivalent (electrode) mercury (Hg^2+^), CA mercuric thiolate HgCA_2_:
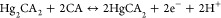
3

In contrast to reactions 1 and 2, where the formation of Hg_2_CA_2_ is faradaic in nature, reaction 3 could be accompanied by a nonfaradaic surface disproportionation of mercurous thiolate into mercuric thiolate and mercury:
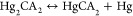
4

Almost the same responses as those produced by CA within the anodic potential region of HMDE were also obtained with CSS (Figure 1A, left). However, prior to oxidation of the electrode mercury, its S–S bond was first reduced (reaction 5), as was indicated by the increase in the cathodic current accompanied by a sharp maximum of the first kind^13^ when the electrode was polarized from −1.0 V in the anodic direction.
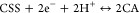
5

Maxima of the first kind, accompanying various reduction and oxidation processes at a dropping mercury electrode, had attracted attention soon after the invention of polarography. Later, they were observed also with HMDE, as well. It is well known that these maxima are caused by streaming of the electrolyte in the vicinity of the electrode, and thus, more electroactive species are transported toward the electrode than by mere diffusion. Questionable is the cause of the origin of the streaming of the solution (for more details, see refs (39−42)).

However, no peak SII and only a poorly developed peak SI were observed in a forward scan of CSS obtained in the cathodic direction (Figure 1A, right). This could be used for discriminating between the CA and CSS in a mixture. Furthermore, processes of S–S bond reduction (reaction 5) and electrode mercury oxidation (reactions 1 and 2) are necessarily separated by the p.z.c., and thus, they could be used to estimate it.

Under the given conditions (pH 6), CA exists in solution in its fully protonated form (Figure 1B). For more details about CA and CSS ionization, see Figures S1, S2 and Table S1 in the Supporting Information. In both CA thiolates, mercury ions can only be bound covalently *via* sulfur atoms. Besides this, nitrogen-containing imidazole^43^ or amino^16,38^ groups have also been found to chelate electrode mercury. This can be true for the CA nonprotonated amino group at approximately pH > 10 (Figure 1B, gray lines). With increasing pH, peaks SII and SI observed in the forward cathodic scan shifted toward more negative potentials. In contrast to peak SII, which decreased steeply between pH 2 and 7, peak SI only changed a little. At pH > 7, peak SII decreased but peak SI increased, both with a sigmoidal course. These significant differences in the heights of peak SI and SII, particularly in alkaline media, can be attributed to the nonfaradaic transition of adsorbed mercuric thiolate of CA into its mercurous thiolate (reaction 4). Thus, peak SI can provide more relevant quantitative information about chemisorbed CA molecules than peak SII. Peak SI corresponds to the so-called “peak S” observed in Cys-containing peptides and proteins.^44^

**Figure 2:**
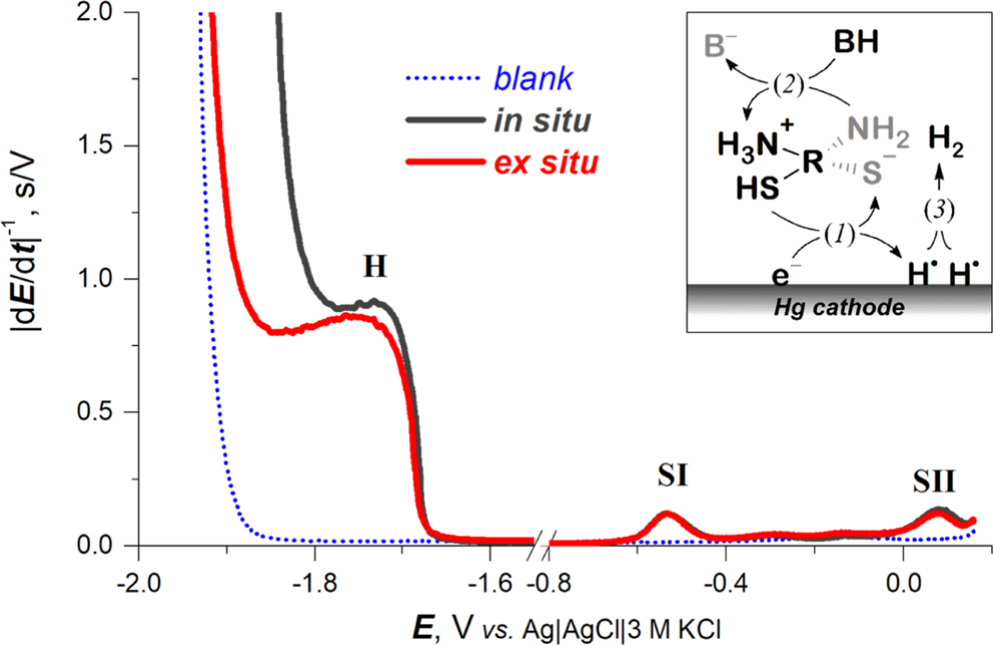
Conventional (*in situ*) and AdT (*ex situ*) CPS responses of 10 μM CA at HMDE in 0.15 M Na-phosphate buffer, pH 7.7. Recorded at −10 μA stripping current from 0.16 V after 60 s of accumulation at open-circuit potential. For more details about AdT-CPS measurement, see the Experimental Section. Inset: Simplified schematic of the involvement of CA in CHER, showing (1) the electrolytic reduction of a proton from the ammonium and thiol groups, which are (2) immediately reprotonated by an excess of a solution acid constituent BH, and (3) molecular gaseous hydrogen formation from the two neighboring surface-bound hydrogen atoms H^•^. For more details about CHER, see ref (48).

We were also interested in the involvement of CA in CHER (inset in Figure 2) that can be summarized by the following irreversible reaction:
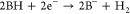
6

Using CV, only a more or less pronounced shift in limiting catalytic currents due to hydrogen evolution (known as presodium shift)^45^ was observed, depending on pH (inset in Figure 1C). Plotting the differences between the *E*_b_ and *E*_H_2__ values showed an optimum pH for CHER at 7.7 (Figure 1C, black spheres). In comparison to CV, CPS is more suited for the measurement of CHER, particularly due to the different mode of electrode polarization.^17^ This enabled us to follow peak H, corresponding to the CHER, and peaks SII and SI in a single (cathodic) scan (Figure 2).

In addition to conventional *in situ* measurement, an *ex situ* adsorptive transfer (AdT) technique^46^ was also used. Peaks SII and SI practically did not differ between the *in situ* and *ex situ* experiments (Figure 2). Peak H was much better developed using the latter because there is no contribution of CA diffusion from the bulk into the interface during the CPS scan. The dependence of the areas of peaks SI, SII, and H on accumulation time (*t*_A_) showed that conditions suggesting full electrode coverage by CA molecules were attained at a *t*_A_ of 60 s (Figure S3 in the Supporting Information). The level of condensation of chemisorbed CA molecules was tested using a Ru(NH_3_^+^)_6_Cl_3_ probe^47^ in weakly acidic and alkaline media, where CA behaves as a monodentate and bidentate ligand, respectively. The CA adsorbate did not affect the electron transfer of the Ru probe redox couple.

The formation of compact impermeable chemisorbed monolayers on the positively charged surface of mercury electrode by SH-group-containing compounds *via* the Hg–S-bond, such as e.g., self-assembled-monolayers of various alkanethiols,^47^ is conditioned particularly by favorable mutual lateral interactions between the anchored molecules. In the case of CA molecules, under conditions shown in Figure 2, only its negligible 1% fraction with the (deprotonated) NH_2_-group is present in solution (Figure 1B). Hence, (i) strong electrostatic repulsion between (protonated) H_3_N^+^-groups along with (ii) only a small contribution of hydrophobic interactions between short aliphatic chains –CH_2_–CH_2_– of neighboring CA molecules play the decisive role. This statement is in good agreement with the CA adsorption/desorption behavior in the HMDE|solution interface followed by phase-sensitive ACV under conditions shown in Figure 2. Even though both the underlying faradaic processes of CA or CSS (peaks SI and SII, Figures 1A and 2) are accompanied by a major change of the electrode capacity, no signs of appreciable adsorption of the chemisorbed positively charged CA molecules on the positively charged electrode surface were detected (not shown). The same was true for the negatively charged interface.

To support the experimental data, we performed MD simulations and QM calculations with CA molecules bound to the previously parametrized mercury surface.^21^ The role of protonation states of the CA amino group, surface densities, and applied voltages was considered. Computational setups are shown in Scheme 1. The setup corresponds to the behavior of chemisorbed CA in the form of mercurous thiolate (peak SI in Figures 1A and 2).

**Figure 3:**
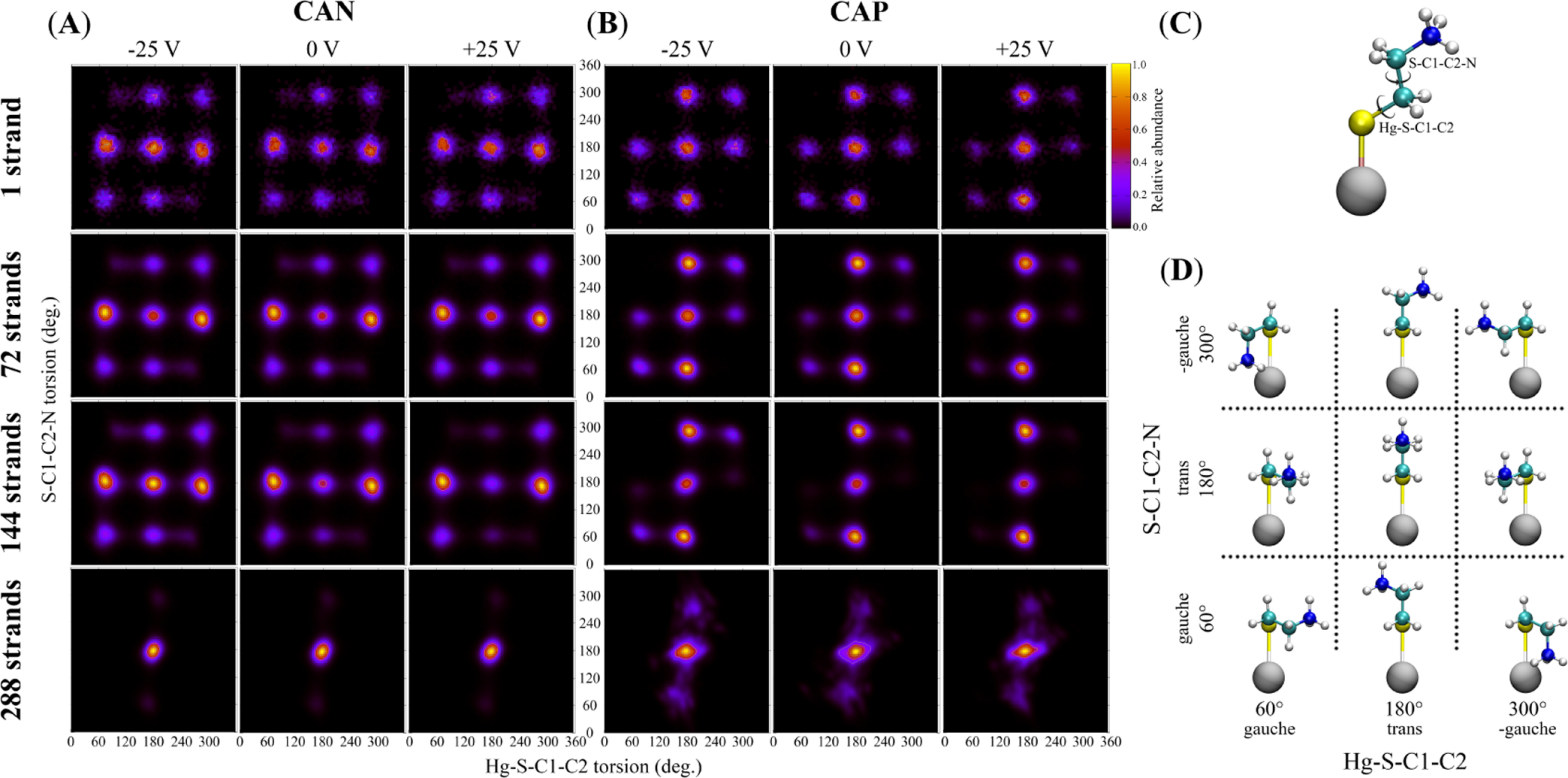
Distribution of torsion angles Hg–S–C1–C2 vs S–C1–C2–N at different surface densities of CA bound to differently charged mercury surface. (A) Neutral CA (CAN), (B) charged CA (CAP), (C) definition of torsion angles, and (D) CA conformers. For the definition of variables, see Scheme 1.

Isolated CA, being a system with no multiple bonds, is flexible, and there are a variety of almost isoenergetic conformers (Figure S4 in the Supporting Information). The presence of additional mercury atoms in the vicinity of CA leads to a certain restraint of free rotation around the single bonds of this molecule. The conformational behavior of CA molecules bound to a charged or uncharged mercury monatomic layer based on the distribution of CA torsion angles Hg–S–C1–C2 vs S–C1–C2–N is visualized in Figure 3. In the presence of an isolated CA molecule (1 strand) and low surface coverage (72 strands), the protonated (CAP) and neutral (CAN) CA forms exhibit differences only for the most abundant conformations (the brightest spots in Figure 3). In particular, the different distribution of highly abundant CAP compared to CAN conformers is reflected in the average distance of their N atoms from the mercury surface (Figure S5 in the Supporting Information). CAN molecules, which exhibit a bimodal distribution, tend to be oriented more in parallel with the mercury surface compared to CAP molecules, with a unimodal distribution with almost perpendicular orientation. This behavior is supported by QM calculations utilizing a cluster of nine mercury atoms, which show the disadvantage of the ±*gauche*/*trans* arrangement for the CAP form (Table S2 in the Supporting Information). The different affinities of ammonium and amino groups to the mercury cluster as well as the mutual intermolecular repulsion of the ammonium groups at higher CA coverages should also be considered. The low abundance of the *gauche*/*-gauche* and *-gauche*/*gauche* conformers is a common denominator for all of the cases shown in Figure 3. According to the QM calculation, such conformers are energetically unfavorable due to steric hindrance (Table S2 in the Supporting Information).

As the mercury surface becomes more covered (144 strands), significant changes in the distribution and abundance of conformations occur mainly for the CAP form. The occurrence of structures that occupy the largest area relative to surface (±*gauche*/*trans*) is suppressed. Conversely, the occurrence of the stretched conformations *trans*/± *gauche* is increased. The maximum surface coverage of 288 strands only allows the existence of the least bulky *trans*/*trans* conformation for both the CAN and CAP forms, although this conformation is itself energetically unfavorable.

It is clearly visible in Figure 3 that CAP molecules are more sensitive to the electric field than CAN molecules. The ±*gauche/*±*gauche* conformation, which facilitates the interactions between the positive ammonium group and the negatively charged surface, tends to vanish upon changing the applied negative voltage to a positive voltage.

## Conclusions

We found that CA forms chemisorbed layers on the surface of the mercury electrode. The formation of Hg-CA complexes is connected to mercury disproportionation, as reflected in peaks SII and SI. Both the process of chemisorption of CA and its subsequent redox transformation are proton-dependent. Also, depending on the protonation of CA, the formation of typical populations of chemisorbed conformers can be observed. In addition, CSS can be reduced on the mercury surface. Between the potentials of this reduction and peak SI, the p.z.c. of the electrode used can be found. Furthermore, CA can serve as an LMW catalyst of hydrogen evolution. In this sense, we expect considerable potential for the application of a CA-modified mercury electrode for probing solvation and hydration processes. The mechanistic insights presented here can be used for follow-up research on CA chemisorption and the targeted modification of other metallic surfaces.

The *in silico* approach helped to investigate the intricate conformational behavior of CA molecules when attached to a mercury surface under varying conditions, considering the factors of charge state, surface density, and applied voltage. These findings shed light on the different preferences of the CAN and CAP forms in these complexes, offering valuable insights for understanding molecular interactions at the nanoscale.

## Supporting Information Available

Acid–base properties of CA and CSS (Figures S1 and S2); macro- and micro-dissociation constants (Table S1); chronopotentiometric stripping of CA; effect of time of accumulation (Figure S3); computational results; surface potential energy of CA bound to mercury atom (Figure S4); average height of N atom(s) of CA over mercury surface (Figure S5); and relative energies of different conformers of CA covalently bonded to mercury (Table S2) (PDF)
