# Supplementary material for: Cysteamine Chemisorption at Mercury–Solution Interfaces in the Context of Redox and Microdissociation Equilibria
Source: Langmuir. 2024 Mar 15;40(12):6253–60. doi: 10.1021/acs.langmuir.3c03744 (PMC10976880; doi:10.1021/acs.langmuir.3c03744)
Supplement: Supplementary file 1 — la3c03744_si_001.pdf [file la3c03744_si_001.pdf]

Supporting Information

Cysteamine Chemisorption at Mercury-Solution Interfaces in the Context of Redox and Microdissociation Equilibria

Vlastimil Dorčák,[1] Ondřej Kroutil,[2] Martin Kabeláč,[3] Jiří Janata,[4] Jan Vacek[1,*]

[1]Department of Medical Chemistry and Biochemistry, Faculty of Medicine and Dentistry, Palacky University, Hnevotinska 3, 77515 Olomouc, Czech Republic

[2]Central European Institute of Technology, Masaryk University, Kamenice 5, Brno, 625 00, Czech Republic

[3]Department of Chemistry, Faculty of Science, University of South Bohemia, Branisovska 31, 370 05 Ceske Budejovice, Czech Republic

[4]School of Chemistry and Biochemistry, Georgia Institute of Technology, Atlanta, 30332-0400, GA, United States

[*]E-mail: jan.vacek@upol.cz

Content: **Page**

Acid-base properties of cysteamine and cystamine	S2
Chronopotentiometric stripping of cysteamine	S3
Computational results	S4

Acid-base properties of cysteamine and cystamine

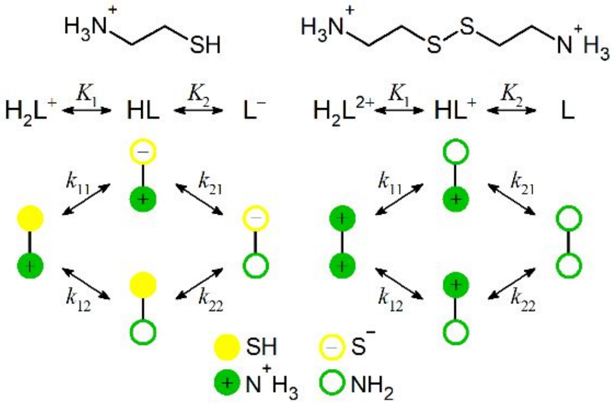

Figure S1. Structures of fully protonated molecules of cysteamine (CA, left) and cystamine (CSS, right) with corresponding macro- and microdissociation schemes.

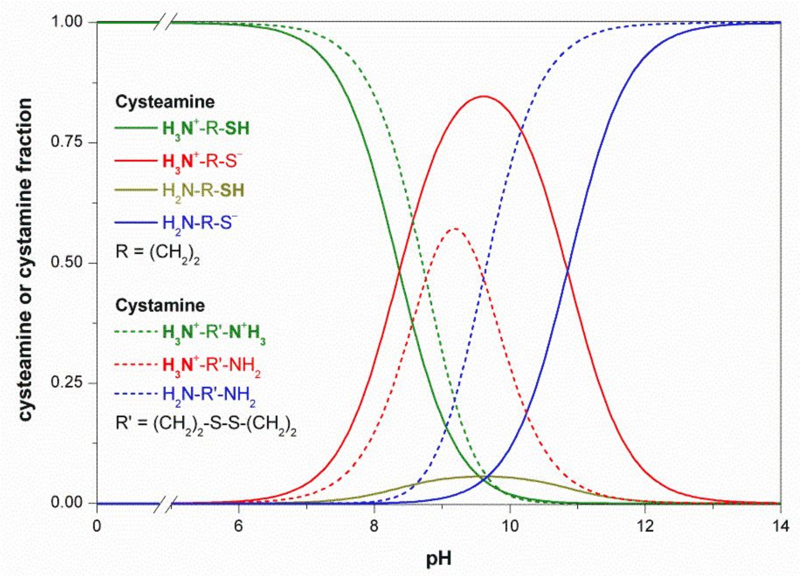

Figure S2. Speciation diagram of CA and CSS (solid and dashed lines, respectively) calculated from microdissociation constants listed in Table S1.

Table S1. Macro- and microdissociation constants of CA and CSS at ionic strength of 0.15 M taken from ref. [A. Mirzahosseini and B. Noszál, *J. Pharm. Biomed. Anal.*, **95** 184-192 (2014)].

CA		CSS	
pK_1 = 8.34	pK_2 = 10.88	pK_1 = 8.76	pK_2 = 9.61
pk_{11} = 8.37	pk_{21} = 10.85	pk_{11} = pk_{12} = 9.06	pk_{21} = pk_{22} = 9.31
pk_{12} = 9.55	pk_{22} = 9.67		

Chronopotentiometric stripping of cysteamine

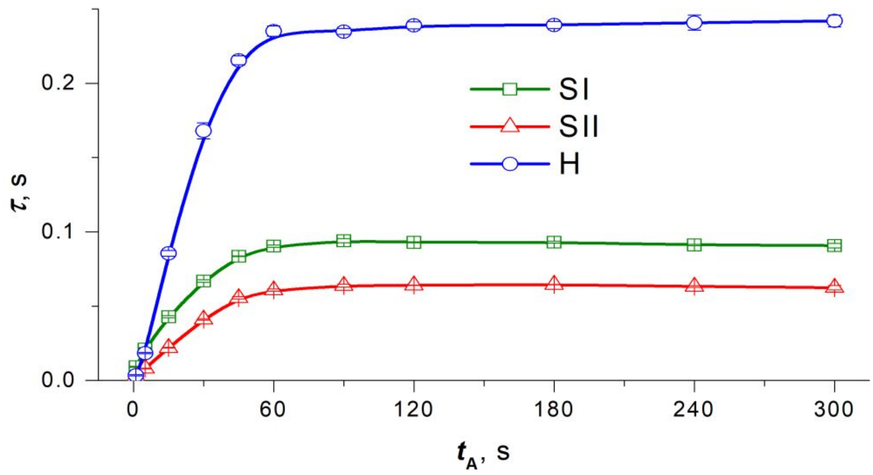

Figure S3. t_A-dependences of *in situ* CPS peak SI, SII, and H area (equal to transition time, τ) of 10 μM CA in 0.15 M Na-phosphate buffer solution of pH 7.7. Read from chronopotentiograms recorded from 0.16 V, after accumulation at E_A of 0.16 V with an I_{str} intensity of –1 μA.

Computational results

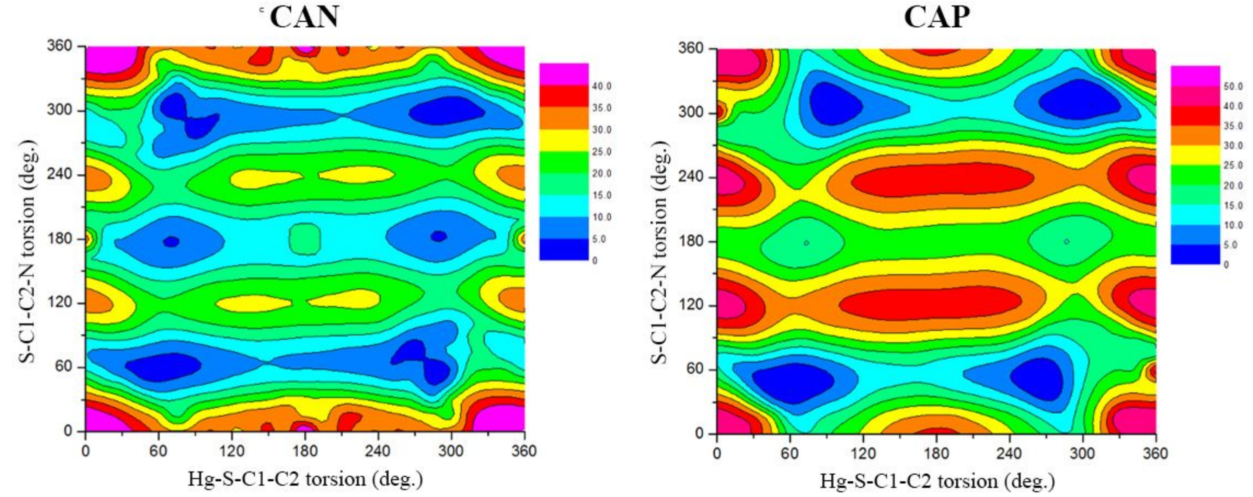

Figure S4. Surface potential energy of cysteamine bound to a single mercury atom represented as two-dimensional dihedral profile of Hg-S-C1-C2 *vs.* S-C1-C2-N torsion angles. Relative energies are given in kJ/mol. The left image corresponds to the non-protonated form of cysteamine (CAN), the right to the protonated form (CAP).

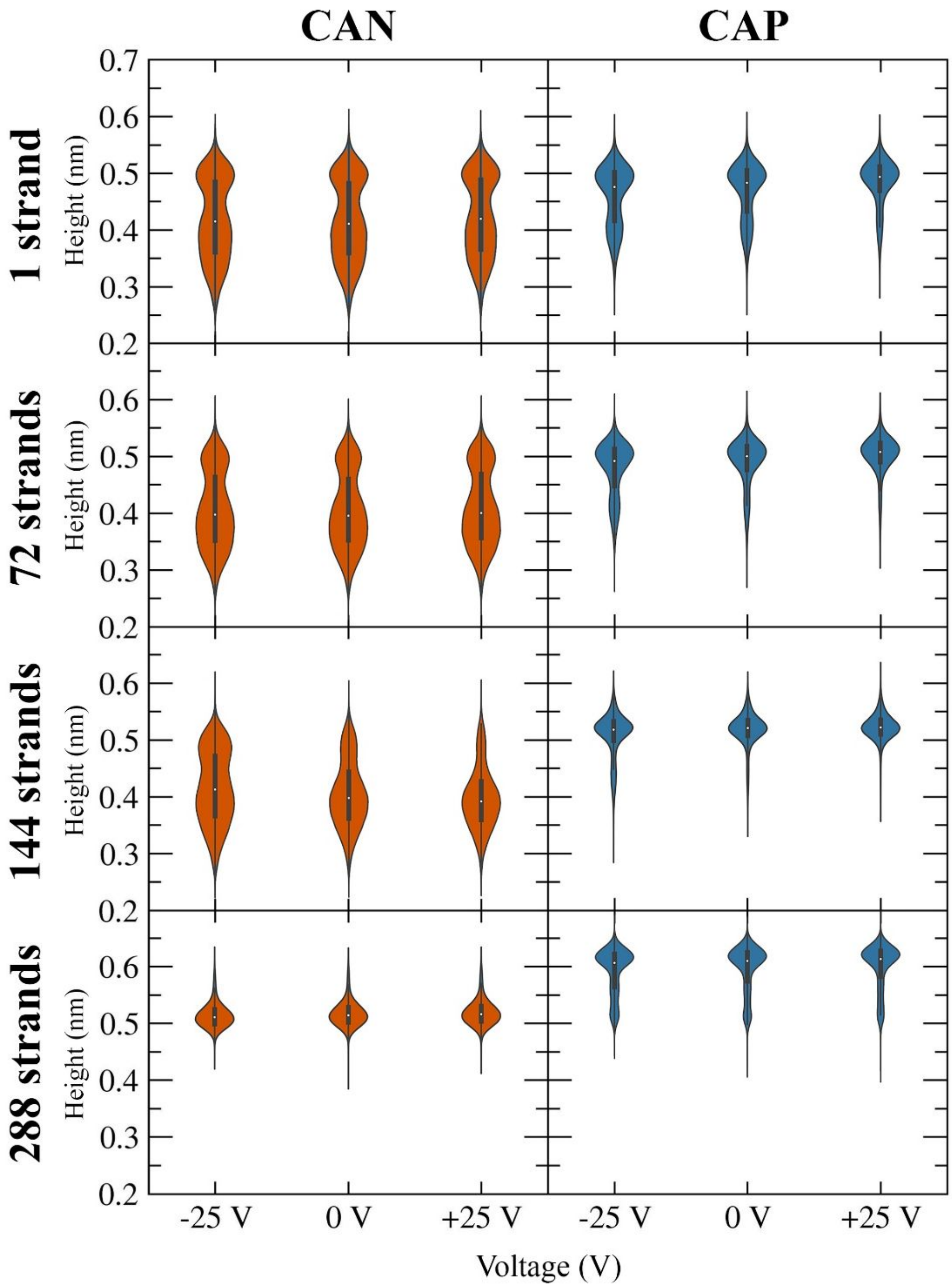

Figure S5. Boxplots displaying average height of N atom(s) over mercury surface for different voltages, coverages and cysteamine protonation state. Dark rectangle represents data between 25th and 75th percentile with the median in the middle. The left image corresponds to the non-protonated form of cysteamine (CAN), the right to the protonated form (CAP).

Table S2. Relative energies (in kJ/mol) of different conformers of cysteamine covalently bonded to mercury. Notable energetic differences in system with cluster of nine mercury atoms are highlighted in colour.

**Torsion (deg.)**		**Cysteamine + 1 Hg**		**Cysteamine + 9 Hg***	
**Hg-S-C1-C2**	**S-C1-C2-N**	**CAN**	**CAP**	**CAN**	**CAP**
gauche	*gauche*	0.0	0.0	3.9	5.5
gauche	*trans*	2.8	11.7	8.3	19.0
gauche	*-gauche*	2.5	10.9	**31.4**	**33.8**
trans	*gauche*	7.1	8.1	12.2	10.3
trans	*trans*	17.4	19.4	9.4	10.9
trans	*-gauche*	7.1	8.1	9.2	10.8
-gauche	*gauche*	2.5	10.9	**33.2**	**36.2**
-gauche	*trans*	2.8	11.7	3.0	18.7
-gauche	*-gauche*	0.0	0.0	0.0	0.0

*Since the system has no symmetry anymore, the ±*gauche* conformations are no longer isoenergetic.
